# Knowledge and Awareness of Syncope Among General Populations of Makkah Region in Saudi Arabia: A Cross-Sectional Study

**DOI:** 10.7759/cureus.38276

**Published:** 2023-04-29

**Authors:** Rawan Aljuwaybiri, Fatima A Almekhlafi, Rawan M Alzahrani, Reham T Almehmadi, Marwah Y Alsubhi, Asayel T Alruwais, Mokhtar M Shatla

**Affiliations:** 1 Medicine and Surgery, Umm Al-Qura University College of Medicine, Makkah, SAU; 2 Family and Community Medicine, Umm Al-Qura University College of Medicine, Makkah, SAU

**Keywords:** saudi, makkah, population, knowledge, level, syncope

## Abstract

Background

Syncope is a transient loss of consciousness and postural tone due to global cerebral hypoperfusion which is followed by spontaneous recovery. It is relatively common and increases the risk of subsequent falls and injury. There is scant literature and targeted research on the population’s knowledge of syncope. Hence, this study aimed to assess awareness and evaluate the knowledge of syncope among the population of the Makkah region.

Methodology

An online cross-sectional study was done on 563 participants. A questionnaire was used to collect data about participants' demographics, experiencing syncope in relatives, and knowledge about syncope using case scenarios.

Results

Females represented 72.6% of the participants and about one third of participants admitted that they had experienced syncope throughout their life. About one-third of participants admitted that they had experienced syncope throughout their life. Most of the study participants (68.9%) showed a good level of knowledge about syncope while 31.1% of them had poor knowledge; the average awareness score was 5.3±1.64. Cardiogenic syncope was the most commonly recognized type of syncope. Furthermore, participants aged between 18 and 35 years and widowed participants demonstrated a good amount of knowledge about syncopal attacks (p<0.001).

Conclusions

General population of the Makkah region in Saudi Arabia had a sufficient level of knowledge about syncope. Additional studies along with educational programs are needed.

## Introduction

Syncope is an abrupt, brief loss of consciousness and postural tone due to global cerebral hypoperfusion followed by spontaneous recovery [1]. Approximately 40% of the population has experienced syncope at least once in their lifetime, making it a prevalent clinical condition [2].

Syncope affects the general population with a prevalence of 42%, an annual incidence of 6%, and several episodes ranging from 18.1 to 39.7 per 1,000 patients [3]. In general, the first peak of incident occurs at the age of 15 years, mainly of vasovagal syncope, and an additional peak after the age of 65 years, the incident rises significantly by the age of 70 years, from 5.7 episodes per 1,000 person per year in men of 60-69 years to 11.1 episodes in men of 70-79 years [4]. Furthermore, syncope is a common medical emergency with an admission rate of 32%, which accounts for 2% of all emergency department admissions [5]. The most prevalent medical emergency reported in dental practices is syncope [6].

Recurrent syncope occurs in about one-third of the patients in the general population. Syncope increases the risk of subsequent falls and injury [7]. The annual mortality in cardiac syncope is around 18-33% and in non-cardiac syncope is around 0-12% [3]. A study that evaluated the knowledge and awareness of syncope among the general population in Saudi Arabia from Riyadh showed that 55% of participants were aware of syncope. Among the causes of syncopal loss of consciousness, 79% of participants were aware of orthostatic syncope and 61% were aware of vasovagal syncope [5].

Most syncopal events are benign causes, which include volume depletion, vasovagal (neurocardiogenic), or medication-related causes. More concerning causes include dysrhythmia and valvular abnormalities including ventricular tachycardia, atrioventricular (AV) block, or aortic stenosis [1]. The most prevalent type of syncope in adults is vasovagal syncope. It is responsible for more than 85% of syncopal episodes in people under 40 years of age. In elderly patients, more than 50% of syncopal episodes are caused by vasovagal syncope. Vasovagal syncope (VVS) is a form of reflex syncope in which there is a failure in blood pressure and cerebral perfusion pressure autoregulation. The mechanisms underlying this involve a decrease in cardiac output in addition to a decrease in vascular tone. This starts with a trigger, which is usually in combination with central hypovolemia brought on by an upright posture or dehydration causing an increase in cardiac contractility in the context of underfilled left ventricle. This will stimulate mechanoreceptors in the ventricle to interact with the central nervous system via vagal afferents, leading to a decrease in the heart rate because of increased parasympathetic activity. Vascular tone is also decreased as a result of decreased sympathetic activity. And because of cerebral autoregulation, cerebral blood flow remains constant at a variety of mean arterial pressures, but the patient loses consciousness when the mean arterial pressure drops below the body's ability for autoregulation [8].

Syncope is relatively common, however, there is scant literature and targeted research on the population’s knowledge of syncope. Hence, this study aimed to assess awareness and evaluate the knowledge of syncope among the population of Makkah region [9].

## Materials and methods

Study design and setting

A cross-sectional study was conducted over four months from December 2022 to March 2023. Makkah region includes 11 governorates, mainly (Makkah, Jeddah, and Taif). A pre-designed questionnaire based on a previous study was used to collect the research data [5]. The Biomedical Research Ethics Committee of Umm Al-Qura University reviewed and approved the study.

Study sample

The sample size for this study was calculated to be 384 participants using OpenEpi version 3.0 [10] based on the size of the total population in the Makkah region [11]. The total population was 8,557,766,2 at a 95% CI and a 5% margin of error. Nevertheless, we included more responses until we reached 756 participants to make the study more meaningful. However, a total of 193 responses were excluded for unmatched inclusion criteria (refusal to participate, all health care practitioners, <18 years of age). The remaining 563 responses were included in the analysis. The inclusion criteria were participants aged 18 years and above, of both genders, Saudi and non-Saudi participants from Makkah region.

Data collection

A self-administered questionnaire was used in a Google form and distributed via WhatsApp groups, and Telegram groups. The questionnaire consisted of three sections. The first section included items about participants' demographics (age, gender, nationality, educational level, marital status, history of chronic disease, and having a professional medical background). The second section was about the history of syncope among participants' relatives. The last section assessed participants' knowledge about syncope by displaying eight case scenarios to evaluate the ability of respondents to recognize the presentation of syncope. The first, second, and fourth cases represented a type of syncope (vasovagal, cardiogenic, and orthostatic hypotension), whereas the third, fifth, sixth, seventh, and eighth cases represented other causes of loss of consciousness (hypoglycemia, coma, pre-syncope, alcohol intoxication, and epileptic attack). Participants had to answer and identify each scenario as either a presentation of syncope or not. Participants who correctly identified five or more out of the eight cases were considered aware of syncope, and those who identified less than five cases were considered unaware of syncope.

Data analysis

For data analysis, SPSS version 23 (Armonk, NY: IBM Corp.) was used. Descriptive statistics were obtained to summarize data, synthesize and report the variables. In which, numerical data were presented as mean±SD. For categorical variables, percentages and frequencies were used. Chi-squared test and Fisher's exact test were used for the association between categorical variables. A p-value of less than 0.05 was considered statistically significant.

## Results

Socio-demographic characteristics of the study respondents

This study included a total of 563 participants, female respondents represented the majority of study population 409 (72.6%). Most of the participants, 355 (63.1%), belonged to the age group of 18-35 years, and the majority of them were Saudi Arabian 523 (92.9%). Regarding the educational level of respondents, we found that the highest proportion of them, 408 (72.5%) participants, had a university degree. More than half of the participants, 290 (51.5%), were married whereas 249 (44.2%) were single. Our results revealed that 439 (78%) participants did not have any history of chronic disease. However, 42 (7.5%) had a history of diabetes mellitus, 38 (6.7%) mentioned hypertension, and only eight (1.4%) had experienced heart disease. Only 90 (16%) respondents had a professional medical background. Furthermore, about one-third of the study population, 186 (33%) participants, admitted that they had experienced syncope before, and nearly half of them, 284 (50.4%) participants, had a related person who experienced a syncope (Table 1).

**Table 1:** Demographic data of the study respondent (n=563).

Variable	Categories	Frequency	Percent (%)
Age (in years)	18-35	355	63.1
	36-50	158	28.1
	>50	50	8.9
Gender	Male	154	27.4
	Female	409	72.6
Nationality	Saudi	523	92.9
	Non-Saudi	40	7.1
Level of education	Fundamental education	127	22.6
	University degree	408	72.5
	Higher education (postgraduate degree)	28	5
Marital status	Single	249	44.2
	Married	290	51.5
	Divorced	18	3.2
	Widowed	6	1.1
History of chronic disease	None	439	78
	Heart disease	8	1.4
	Hypertension	38	6.7
	Diabetes mellitus	42	7.5
	Other	51	9.1
Do you have a professional medical background?	Yes	90	16
	No	473	84
Have you ever experienced syncope?	Yes	186	33
	No	377	67
Have someone related to you experienced any type of syncope?	Yes	284	50.4
	No	279	49.6

**Figure 1:**
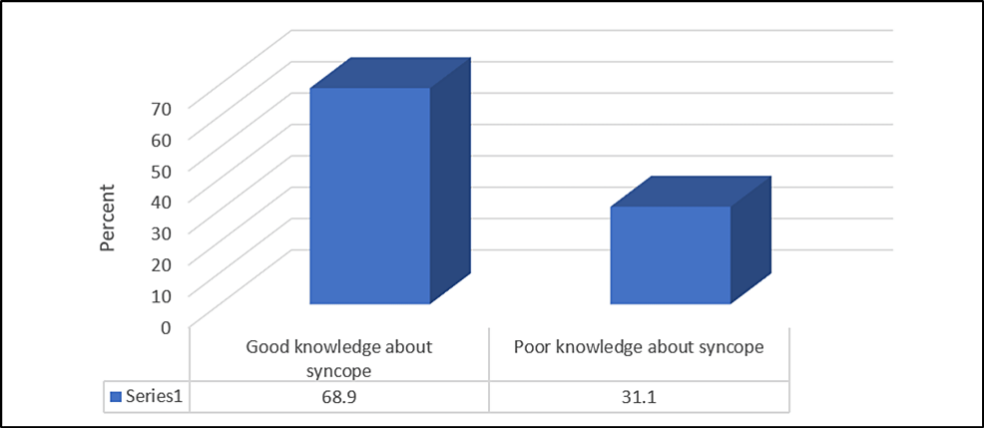
Level of knowledge and awareness of syncope.

Our findings demonstrated that most of the study participants, 388 (68.9%), showed a good level of knowledge about syncope (scored 5 or more) while 175 (31.1%) participants had poor knowledge (scored less than 5). The average awareness score was 5.3±1.64 (1-8) out of 8 (Figure 1).

Case scenarios

Our study displayed the below eight case scenarios to evaluate the ability of respondents to recognize the presentation of syncope. We found that the majority of participants, 469 (83.3%), identified scenario number two which represented a cardiogenic syncope. Followed by the scenario of orthostatic hypotension syncope (number four) which was recognized by 433 (76.9%) participants. The vasovagal syncope in scenario number one was identified by more than two-thirds of respondents, 375 (66.6%).

Additionally, scenario number seven which is about alcohol intoxication yielded 421 (74.8%) correct answers. A total of 391 (69.4%) participants revealed the right answers regarding scenario number six about coma after road traffic accidents. A total of 414 (73.5%) participants correctly answered scenario number five. Less than half of the respondents, 268 (47.6%), revealed the correct answer about the hypoglycemic coma in scenario number three. Only 208 (36.9%) participants correctly answered scenario number eight about a seizure attack (Table 2).

**Table 2:** Distribution of the study participants according to their responses to the case scenarios. *Syncopal causes.

Scenarios	Syncope, n (%)	Not syncope, n (%)
1. A student in primary school lost consciousness after she saw the needle for her common flu vaccination.*	375 (66.6)	188 (33.4)
2. Your neighbor, who is suffering from cardiac disease, suddenly fell unconscious while praying. However, he gained consciousness immediately.*	469 (83.3)	94 (16.7)
3. A person with diabetes, who did not eat breakfast this morning, presented in front of a group. He felt dizzy and his heart was racing.	295 (52.4)	268 (47.6)
4. An elderly man, who takes medication for his hypertension, was lying down. Suddenly, he stands up and then feels unsteady and falls.*	433 (76.9)	130 (23.1)
5. A woman with anemia stood up suddenly and felt dizzy. She was going downstairs and was about to fall, but she held on to the handrails and managed to steady herself.	149 (26.5)	414 (73.5)
6. After having severe injuries from a car accident, a patient was taken to the hospital and was unconscious until he finally woke 10 days later.	172 (30.6)	391 (69.4)
7. A person under the influence of alcohol felt tipsy and fell.	142 (25.2)	421 (74.8)
8. A person in a supermarket started to feel dizzy, and after a while, he fell unconscious. After the fall, he started to bite his tongue and have convulsions. After 10 minutes, he regained consciousness.	355 (63.1)	208 (36.9)

Association between knowledge and awareness of syncope and different demographic factors

Our results showed that participants who belonged to age group 18-35 years and those who were widowed significantly associated with a good knowledge and awareness of syncope (p-values<0.001 for each). Other factors, such as gender, nationality, level of education, history of chronic disease, having a professional medical background, experiencing syncope, and having someone who experienced syncope, did not show any significant association with awareness towards syncope (p-value>0.05) (Table 3).

**Table 3:** Association between knowledge and awareness of syncope and different demographic factors. *P-value<0.05 is considered statistically significant. **P-value calculated using Fisher's exact test. Other p-values calculated using chi-square test.

Variable	Categories	Level of knowledge		p-Value
		Good	Poor	
		n (%)		
Age (in years)	18-35	268 (75.5)	87 (24.5)	<0.001*
	36-50	94 (59.5)	64 (40.5)	
	>50	26 (52)	24 (48)	
Gender	Male	103 (66.9)	51 (33.1)	0.522
	Female	285 (69.7)	124 (30.3)	
Nationality	Saudi	364 (69.6)	159 (30.4)	0.206
	Non-Saudi	24 (60)	16 (40)	
Level of education	Fundamental education	88 (69.3)	39 (30.7)	0.135
	University degree	276 (67.6)	132 (32.4)	
	Higher education (postgraduate degree)	24 (85.7)	4 (14.3)	
Marital status	Single	192 (77.1)	57 (22.9)	<0.001**
	Married	177 (61)	113 (39)	
	Divorced	13 (72.2)	5 (27.8)	
	Widowed	6 (100)	0 (0)	
History of chronic disease	Yes	59 (65.6)	31 (34.4)	0.452
	No	329 (69.6)	144 (30.4)	
Having a professional medical background	Yes	59 (65.6)	31 (34.4)	0.452
	No	329 (69.6)	144 (30.4)	
Have you ever experienced syncope?	Yes	132 (71)	54 (29)	0.460
	No	256 (67.9)	121 (32.1)	
Have someone related to you experienced any type of syncope?	Yes	206 (72.5)	78 (27.5)	0.061
	No	182 (65.2)	97 (34.8)	

## Discussion

The current study aimed to evaluate the general public's knowledge and awareness of syncope in the Makkah region, Saudi Arabia. Syncope is defined as a loss of consciousness caused by a sudden decrease in cerebral blood flow. The most prevalent forms are observed in daily life and are caused by regular physiological mechanisms [12]. Syncope may account for 1-3% of emergency department consults and roughly 1-3% of hospital hospitalizations [13].

The present study reported that most of the study population had a good level of knowledge about syncope. Comparatively, a study carried out in the Saudi Arabian city of Riyadh revealed that 55% of participants had knowledge of syncope [5]. Moreover, a study conducted in Saudi Arabia revealed a lower level of awareness than our findings, which indicated that only 39.5% of participants recognized syncope or dizziness [14]. However, a different survey among Indonesian schoolchildren found that 97.5% were at a good level, while only one person, or 2.5%, was at a poor level [15]. These variations between the studies are most likely due to differences in the scoring system and features of sampled populations.

Our results reported that about one-third of the study population had experienced syncope before. This was higher than another previous study which showed that about 20% of people have experienced syncope at least once in their lives and 10% of them have experienced it more than once [16].

In this study, the majority of participants identified scenario number two which represented a cardiac syncope. Followed by the scenario of orthostatic hypotension syncope. This contradicted another study conducted in Riyadh, which found that orthostatic syncope had the most correct replies, followed by vasovagal syncope [5]. In the elderly, cardiac causes of syncope, orthostatic hypotension, and postprandial hypotension are more common [17]. This is due to decreased effectiveness of cardiovascular regulatory systems, drug effects compromising orthostatic blood pressure management, and an increase in the prevalence of organic disease (structural heart disease, cardiac arrhythmias, and carotid-sinus syndrome) [18].

When data from specialized cardiac syncope units is examined, cardiac reasons become even more apparent. According to several studies, 45-80% of unexplained syncope in this setting can be attributed to a cardiac etiology [19]. In this study, only 36.9% of participants correctly answered scenario number eight about an epileptic attack. A previous study confirmed this, demonstrating that there is substantial evidence that syncope is frequently misinterpreted as epilepsy [20]. Furthermore, this study showed that age and marital status significantly influenced the level of knowledge of our participants. A previous study in Riyadh reported that male participants who had medical backgrounds were associated with a better level of knowledge about syncope, which was not obtained in our study [5].

Limitations

Our study has some limitations. The proportion of participants with medical backgrounds could contribute to the overestimation of our results. Also, confusion/misunderstanding between syncope and pre-syncope is another concern.

Recommendations

We suggest that obtaining information regarding syncope knowledge, in conjunction with information about its triggers, might serve as a baseline for developing cost-effective diagnostic procedures in the evaluation of patients with syncope in various clinical settings.

## Conclusions

Our study concluded that the general population of the Makkah region in Saudi Arabia had an adequate level of knowledge about syncope. Age and marital status significantly affected the level of knowledge of our participants. Educational programs regarding syncope knowledge and its triggers are needed to raise the population's awareness. Further studies on larger representative samples are needed.
